# A brief review of recent Charcot-Marie-Tooth research and priorities

**DOI:** 10.12688/f1000research.6160.1

**Published:** 2015-02-26

**Authors:** Sean Ekins, Nadia K. Litterman, Renée J.G. Arnold, Robert W. Burgess, Joel S. Freundlich, Steven J. Gray, Joseph J. Higgins, Brett Langley, Dianna E. Willis, Lucia Notterpek, David Pleasure, Michael W. Sereda, Allison Moore

**Affiliations:** 1 Hereditary Neuropathy Foundation, New York, NY, 10016, USA; 2 Collaborations in Chemistry, Fuquay Varina, NC, 27526, USA; 3 Collaborative Drug Discovery, Burlingame, CA, 94010, USA; 4 Arnold Consultancy & Technology LLC, New York, NY, 10023, USA; 5 Master of Public Health Program, Mount Sinai School of Medicine, New York, NY, 10029, USA; 6 Quorum Consulting, Inc, San Francisco, CA, 94104, USA; 7 The Jackson Laboratory in Bar Harbor, Bar Harbour, ME, 04609, USA; 8 Department of Medicine, Center for Emerging and Reemerging Pathogens, Rutgers University – New Jersey Medical School, Newark, NJ, 07103, USA; 9 Gene Therapy Center and Dept. of Ophthalmology, University of North Carolina at Chapel Hill, Chapel Hill, NC, 27599-7352, USA; 10 Quest Diagnostics, Athena Brand, Marlborough, MA, 01572, USA; 11 Burke-Cornell Medical Research Institute, White Plains, NY, 10605, USA; 12 Department of Neurology and Neuroscience, Weill Medical College of Cornell University, New York, NY, 10065, USA; 13 Department of Neuroscience, College of Medicine, McKnight Brain Institute, University of Florida, Gainesville, FL, 32611, USA; 14 Institute for Pediatric Regenerative Medicine, University of California Davis, School of Medicine, Sacramento, CA, 95817, USA; 15 Department of Neurology, University of California, Davis, School of Medicine, c/o Shriners Hospital, Sacramento, CA, 95817, USA; 16 Department of Neurogenetics, Max Planck Institute (MPI) of Experimental Medicine, Göttingen, 37075, Germany; 17 Department of Clinical Neurophysiology, University Medical Center (UMG), Göttingen, D-37075, Germany

**Keywords:** Charcot Marie Tooth, rare disease, CMT1A, biomarkers, drug discovery

## Abstract

This brief review of current research progress on Charcot-Marie-Tooth (CMT) disease is a summary of discussions initiated at the Hereditary Neuropathy Foundation (HNF) scientific advisory board meeting on November 7, 2014. It covers recent published and unpublished *in vitro* and *in vivo* research. We discuss recent promising preclinical work for CMT1A, the development of new biomarkers, the characterization of different animal models, and the analysis of the frequency of gene mutations in patients with CMT. We also describe how progress in related fields may benefit CMT therapeutic development, including the potential of gene therapy and stem cell research. We also discuss the potential to assess and improve the quality of life of CMT patients. This summary of CMT research identifies some of the gaps which may have an impact on upcoming clinical trials. We provide some priorities for CMT research and areas which HNF can support. The goal of this review is to inform the scientific community about ongoing research and to avoid unnecessary overlap, while also highlighting areas ripe for further investigation. The general collaborative approach we have taken may be useful for other rare neurological diseases.

## Introduction

Several recent reviews have focused on the need for discovery of therapies for rare diseases ^1^ as well as the importance of increased collaboration ^2, 3^. There are few approved treatments for the approximately 7000 rare diseases that affect ~6–7% of the population of the developed world ^1^. Advances in technology are changing how rare diseases are discovered ^4^ and deepening our understanding of them ^5^. While suggestions on how to increase the number of drugs developed for rare conditions have been made ^1, 6, 7^, it is unlikely that we are going to see a dramatic change unless there is a wholesale shift in the process of drug discovery and development, combined with increased collaboration between academics, industry, government labs and research foundations in the rare disease arena. Perhaps one of the ways this situation can best be changed is if patients and advocates work with the scientists they fund to identify the critical issues that need addressing ^4, 8^. This may enable developments that are most likely to show some direct benefit for the disease (and patients) to move research from the lab bench to the clinic.

Some rare diseases have recently been in the spotlight for their ability to inspire the raising of large sums of money, which can hopefully accelerate the search for a cure. Most notable is Amyotrophic Lateral Sclerosis (ALS) which became very high profile in 2014 with the ALS ice bucket challenge ^9^. For several years there has been a considerable discussion in this scientific community about difficulties translating the many preclinical studies for ALS from the mouse to the very costly human clinical trials ^10, 11^. It is now apparent that there are several factors that can impact the success of drug discovery even from the very earliest stages, which can in turn influence the transition of new therapeutics into the clinic. When targeting the development of new therapeutics, it is important to consider problematic issues that have prevented success in other disease areas early in the discovery progress. These broader issues can complicate the discovery of new drugs and include: target validation ^12^, artifacts such as promiscuous compounds in small molecule screens ^13– 15^, false positives ^16^, how liquids are moved ^17^, leaching from plastics used in labware ^18^, compound aggregation ^19^, the solvent effects ^20^, detergent effects ^21^, data reproducibility ^21^, data quality ^22, 23^, data access and standards ^24^, data handling ^25^, and biases introduced by filtering libraries ^26^. Even the recent shift of drug discovery from pharmaceutical laboratories to academic screening centers has issues ^27^ and major gaps have been identified ^28^. Many rare disease foundations or researchers are not even aware of these potential complications, which may ultimately impact the outcome and potential clinical feasibility of the work they fund or pursue, respectively.

We will briefly summarize recent developments in one rare disease, Charcot-Marie-Tooth disease (CMT), and propose further, currently untapped, opportunities as well as ultimately list what we believe should be the priorities for the field. While CMT may not be representative of every rare disease, this example may inspire other groups to consider not only the research they are funding but to go beyond the current dogma and consider what type of research needs to be funded to enable compounds to come to the clinic in the short, mid and long term. Drug discovery is not an overnight process as it usually takes well over a decade to go from a discovery in the laboratory to a product that is approved for clinical use. The success transitioning from each stage is variable for drugs with orphan designation (with phase 1 and phase 2 success rates above average at 86.8 and 70%, respectively, while the phase 3 success rate at 66.9% was comparable with all indications) ^29^. This would suggest that if we could slightly improve the success of each stage we would see far more drugs approved. We hope that by producing such a summary of recent work and priorities, there will be similar collaborative discussions between researchers and patient organizations for other rare diseases.

## Diagnosis and mutations

Our particular focus is on CMT, a rare disease which has multiple genetic causes ^30^ and is classified into nine genetic subtypes (CMT1, CMT2, CMT3, CMT4, CMT5, CMT6, CMTDI, CMTRI and CMTX). Research in many areas is redefining our view of this disease and the complexities involved. CMT affects approximately one in 2500 Americans ^31^, who usually have distally pronounced muscle weakness, resulting in difficulty walking and later also gripping objects. Typically, CMT patients display foot deformities, decreased reflexes, and bilateral foot drop ^32^. Recent efforts and progress on CMT emphasize that we are steadily and impressively improving our understanding of the complex underlying biology. At the time of writing, there were over 3800 papers in PubMed on this disease. There is however no treatment for any of this group of disorders encompassed by CMT for which symptoms usually present in the first two decades of life ^33^, so this presents a huge untapped opportunity to improve the lives of the many patients with this debilitating disease.

Accurate diagnosis of CMT is important if we are to identify patients for future clinical trials with treatments for the disease. Currently a tiered approach to genetic testing is used and recommended by clinicians and relies on nerve conduction velocity assessment, disease inheritance pattern and population frequency ^34^. This approach is however laborious, costly and based on recent studies may be ripe for reappraisal. A study in Norway looked at diagnostic laboratory testing for CMT and the spectrum of gene defects in that country ^35^. In total, 549 patients were studied. Nearly 96% of these patients had mutations in just four genes ( *PMP22, MPZ, GJB1* and *MFN2*) linked to CMT. These genetic findings are in accordance with what has also been observed in other countries. In the United Kingdom a study of 1607 patients showed mutations or rearrangements in the same four genes in over 90% of the samples ^36^. In these two studies, patients without a mutation in these four genes were then considered for referral ^31, 37^. A more recent study in the United States ^30^ has evaluated over 17,000 patients using a variety of gene testing methods. The scale of this study is at least 10 times larger than previous analyses and reproduced the finding that almost 95% of patients had mutations in just four genes. Specifically, it showed that 78.6% of those tested were positive for copy number variations of *PMP22*. The genes *GJB1, MPZ* and *MFN2* were present in 6.7%, 5.3% and 4.3%, respectively. These combined studies point to an opportunity for changing the algorithm for CMT diagnosis with initial focus on testing just these 4 genes, and patients that are negative for these can then be evaluated further with nerve conduction velocity testing. Early diagnosis has important benefits as the downstream costs of not treating CMT are considerable, and it can prevent unknowingly exacerbating the disorder by avoiding drugs that are contraindicated.

New CMT mutations are also continually being sought which may help with diagnosis and biological understanding of the disease. This is particularly important in CMT2, in which, unlike CMT1A, the most common mutations (MFN2 and GDAP1) account for only 25% of the total. Next generation sequencing (NGS) was used to screen CMT2 genes in 15 patients in whom *MFN2* and *GDAP1* mutations were excluded ^38^. A new mutation in *HSPB1* was identified, a c.404C>A transversion resulted in p.(Ser135Tyr) amino acid change. A previous p.(Ser135Phe) *HSPB1* mutant was found to impact cell viability and neurofilament assembly in cultured cell experiments ^38^. It is highly likely that the newly discovered mutation has a similar if not identical role. It was suggested that NGS is a tool for efficient mutation detection and exclusion in CMT2 ^38^.

The role of aminoacyl-tRNA synthetases (ARS) has recently been reviewed as housekeeping enzymes ^39^. That is to say ARS have a key role in ensuring accurate transfer of information in the genetic code. Mutations in *GARS* (glycyl-tRNA synthetase gene), *KARS* (lysyl-tRNA synthetase gene), *AARS* (alanyl-tRNA synthetase gene) and YARS (tyrosyl-tRNA synthetase gene), all are definitively associated in causing axonal CMT ^40– 43^. Furthermore, evidence supports the additional association of lysy’-tRNA synthetase, methionyl-tRNA synthetase and histidyl-tRNA synthetase in peripheral axon degeneration ^44, 45^. For example CMT2D is an axonal neuropathy characterized by a phenotype that is more severe in the upper extremities. Mutations in the gene encoding *GARS* have been implicated in CMT2D. Mutations in *GARS* show loss-of-function features ^46, 47^, suggesting that tRNA-charging deficits play a role in disease pathogenesis, but animal studies support a gain of function mechanism ^47, 48^. Yet the linkage between mutations and subsequent pathology is unclear. It is difficult to reconcile the mutations in these enzymes with CMT, and numerous possible etiologic mechanisms from loss-of-function to gain-of-function including many that may be outside their usual role ^49^. The latter may be outside their usual role. Yao and Fox ^39^ also describe the role of the ARS proteins in other organisms such as yeast and bacteria as well as drug targets (e.g. antitumor). This should be considered in light of possible complications one sees with other chemotherapeutic agents precipitating CMT. This would be a very important consideration if ARS were to be targeted for modulating various diseases and hints at the potential of how biological discoveries in one area like a rare disease can shed light on many other diseases at little extra cost.

## Basic research: recent Charcot-Marie-Tooth preclinical research

The progress of CMT has been described in several steps recently ^50^. Genetic defects in myelinating Schwann cells (e.g. *PMP22* duplication, CMT1A ^30, 51^ or overexpression leads to missorting or the accumulation of these mutated/overexpressed proteins. In addition to subsequent demyelination, the malfunctioning Schwann cells then fail to sustain axonal support which results in progressive axonal and neuronal loss. The clinical phenotype of CMT is ultimately determined by the resulting neurogenic muscle atrophy.

Several recent papers provide some encouraging news in the quest for treatments for CMT1A, which is a primary dysmyelinating disease, and have identified compounds that have reached preclinical or clinical stages ( Table 1). In addition to these, one study described how the CMT1A rat model (in early postnatal development) could be treated with a recombinant human growth factor called neuregulin-1 which controls myelin thickness ^52^. In CMT rats this appears to enhance the reduced signaling of phosphatidylinositol 4,5-bisphosphate 3-kinase (PI3K)–v-Akt murine thymoma viral oncogene homolog 1 (Akt) and lower augmented mitogen-activated protein kinase kinase 1 (Mek)-mitogen –activated protein kinase (Erk), and is able to improve the differentiation of Schwann cells in CMT1A. Thus, neuregulin-1 can counter the effect of PMP22 overexpression on downstream signaling. Neuregulin-1 was found to be less effective in treating older animals. This approach may be useful in children where it could be applied before disease onset. This work opens the door for using compounds that modify signaling pathways and the kinases involved.

A second recent paper used genome editing to create an assay for high throughput screening to expand the targets for drug discovery in CMT1A ^53^. The main result of this work was the identification of the protein kinase C modulator bryostatin ( Table 1) which lowers PMP22 expression. Interestingly this compound was not identified in previous screens by the group which had delivered the proteasome inhibitors such as bortezomib ^54^ ( Table 1). In summary, two independent groups have now focused on the role of kinases in pathways that control PMP22. This work may foster a broader consideration of the many compounds already available that modulate different kinases.

**Table 1.** Compounds tested for CMT 1A (impacting Pmp22) *in vitro* and or *in vivo*. (All structures were extracted from ChemSpider ( www.chemspider.com)).

Compounds	Target and status	Source	Ref.
Vitamin C 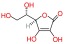	cyclic adenosine monophosphate Reached clinical trials and failed	INSERM	[ 103]
Fenretinide 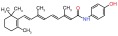	Interaction with retinoic acid receptor and retinoid X receptor Preclinical	NCATS	[ 54]
Olvanil 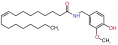	Agonist for the transient receptor potential vanilloid type 1 Preclinical	NCATS	[ 54]
Bortezomib 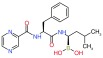	20S proteasome inhibitor Preclinical	NCATS	[ 54]
Bryostatin 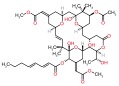	Protein kinase C Preclinical	NCATS	[ 53]
Baclofen 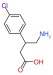 Naltrexone 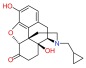 Sorbitol 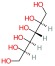	PXT-3003 is a combination of 3 compounds used at lower concentrations than approved Baclofen targets the gamma aminobutyric acid B receptor (GABA(B)), Naltrexone targets opiod receptors, Sorbitol may act as a chaperone and or bind muscarinic acetylcholine G- protein coupled receptors Completed phase 2 clinical trial	Pharnext	[ 90, 91]
ADX71441 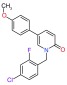	GABA(B) targeted and reduces PMP22 expression Preclinical	Addex Therapeutics	[ 89, 104]
Ethoxyquin 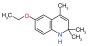	Heat shock protein 90 (HSP90) modulator used to protect against chemotherapy induced neurotoxicity Preclinical	Johns Hopkins University	[ 59]

Alternative approaches in ameliorating the disease phenotype in CMT1A have focused on protein quality control mechanisms, specifically autophagy and chaperones. Rapamycin, a calorie restriction mimetic was shown to improve myelination in neuron-Schwann cell explant cultures from neuropathic mice, however this drug did not improve neuromuscular performance in mice *in vivo* ^55^. The differential response of skeletal muscle and peripheral nerve tissue to rapamycin is hypothesized to be responsible for the lack of functional improvement in neuropathic mice. Additional efforts in identifying therapeutic target pathways for CMT1A neuropathies involve studies on the normal function of PMP22 in myelin. A novel role for PMP22 has been found in the linkage of the actin cytoskeleton with the plasma membrane, possibly through regulating the cholesterol content of lipid rafts ^56^.

Other targets of interest for CMT include histone deacetylase 6 (HDAC6) ^57^ as the inhibition of this can promote survival and regeneration of neurons. It was more recently shown that an increase of α-tubulin acetylation induced by inhibition of HDAC6 corrected axonal transport defects caused by HSPB1 mutations, rescued the CMT phenotype of mutant HSPB1 mice ^58^.

One of the ways that some CMT patients first become aware of their disease is when they are given a drug treatment for another disease. This is termed chemotherapy-induced neurotoxicity. Drugs such as paclitaxel and the vinca alkaloids that are widely used in cancer treatment cause severe peripheral neuropathy and in some patients, this exacerbates CMT, revealing it perhaps for the first time. There have been recent developments in this area that may protect CMT patients in the future. A synthetic antioxidant called ethoxyquin ^59^, which was approved by the US Food and Drug Administration (FDA) 50 years ago, appears to protect against neurotoxicity in both cell and animal studies. This treatment does not appear to impact chemotherapy and also modulated a protein called heat shock protein 90 (HSP90). This work paves the way for further studies of the neuroprotective ability of this and other compounds and possibly clinical trials for patients with pre-existing CMT that need to undergo chemotherapy and for which there are few options. At the same time it may help reveal the mechanisms of neurotoxicity resulting from these chemotherapeutics. A recent study described how inducible HSP70 prevented aggregation and enhanced the processing of PMP22. The authors also proposed the further study of HSP90 inhibitors in models of PMP22-linked neuropathies ^60^.

## Basic research: new animal models

The creation of mouse models for CMT can be used to understand the mechanisms of the various types of this disease and for drug discovery ^61^. Three mouse models of CMT1X, CMT1B and CMT1A were recently developed to study Schwann cells and show that they display a heterogeneous pattern of developmentally regulated molecules ^62^. These could be useful for diagnostic purposes. They also described an inflammatory reaction as a common disease modulator in the mouse and possibly humans with CMT1. LRSAM1 is a E3 ubiquitin ligase and mutations in LRSAM1 have recently been shown to cause CMT. Mouse mutations in Lrsam1 were created for a form of CMT (CMT2P) which had only a very mild neuropathy phenotype with age but was more sensitive to the neurotoxin acrylamide, causing axon degeneration ^63^. LRSAM1 primarily localizes in a perinuclear compartment immediately beyond the Golgi, but its cellular function is not yet understood. However, the phenotype of the Lrsam1 mutant mice is so mild that it is of questionable utility as a disease model, and similar findings in genes such as *Hint1* highlight the challenges of creating valid mouse models of axonal neuropathies ^64^.

While we frequently see groups use mice and rat as animal models of diseases, sometimes they are unsuitable for knocking out genes as they can result in embryonic lethality. The zebrafish has been described as a model of CMT2A which affects the distal axons of motor and to a lesser extent, sensory neurons in humans ^65^. The study assessed the phenotypic effects of mitofusin 2 ( *MFN2*) mutation in zebrafish. The *Mfn2* mutant zebrafish do not develop abnormalities until later stages. Previously it had been shown by others that knocking out the *MFN2* gene in mice resulted in embryonic lethality. Zebrafish created with this mutation in *MFN2* developed normally, however they showed a progressive motor dysfunction as the fish were unable to swim correctly between 100 and 200 days ^65^. Some patients with mutation of the *MFN2* gene also show this progressive motor dysfunction. Fishes were monitored in the study ^65^ by video in their aquarium and those swimming at an angle of more than 30 degrees to the horizontal were specifically recorded ^65^. In addition to these studies, *in vitro* cell culture was utilized to measure the mitochondrial transport in the neurons from the *MFN2* knock out zebrafish and it was found that retrograde transport was decreased. Obviously, while humans and zebrafish appear dissimilar, their MFN2 proteins are very similar. This study therefore presents a potentially very useful animal model of CMT2A that can then be explored to test potential future drugs for their effect on mitochondrial dynamics and axonal transport in order to see if they can ameliorate the phenotype (swimming at an angle of more than 30 degrees) observed.

When we think of the different animal models for diseases like CMT, the fruit fly ( *Drosophila melanogaster*) is likely the furthest from our mind. Yet the fruit fly has quite possibly overturned our basic understanding of the disease mechanism behind CMT2B, by becoming the first animal model for this disease. CMT2B was long thought to be a “gain of function” disease. A recent study however showed that neurons lacking a gene for *rab7* result in neuropathy, while addition of Rab7 proteins could restore function ^66^. Fundamental insights like this can affect how we approach a treatment or cure for CMT2B. Perhaps experimental methods to increase Rab7 protein levels, while opposite to the previous dogma, may actually be correct. There are still multiple hurdles to overcome because the fruit fly may not be a perfect disease model for human CMT2B. A second paper also suggests that the human Rab7 mutation mimics CMT2B in the fly ^67^. In this study, a second fly model was developed and demonstrated attributes of the human disease. Flies have also been successfully used to model neuropathy associated with tRNA synthetases and to identify genetic modifiers of these diseases ^68, 69^. This model could be useful for screening compounds as potential therapies and understanding of the mechanism of the disease. This also represents another case of flies being useful of humans. The increasing utilization of different animal models of various CMTs suggests that their role will likely increase in importance and may lead to useful insights and help identify therapeutics.

## Basic research: gene therapy and stem cells

There have been relatively few studies assessing gene therapy for CMT. One group recently used neurotrophin-3 ( *NT-3*) gene therapy via adeno-associated virus (AAV) delivery to muscle ^70^. In the Trembler-J model of demyelinating CMT, this gene therapy led to measurable NT-3 secretion and improved motor function, histopathology, and electrophysiology of peripheral nerves.

Other hereditary neuropathies have also been the subject of gene therapy. For example Giant axonal neuropathy (GAN) is caused by loss of function of the gigaxonin protein. In cells this is seen as intermediate filament (IF) aggregation, and leads to a progressive and fatal peripheral neuropathy. GAN mice received an intracisternal injection of an AAV9/GAN vector to globally deliver the GAN gene to the brainstem and spinal cord. These mice showed clearance of peripherin IF accumulations suggesting that gigaxonin gene transfer can reverse the pathology ^71^. Other dominant CMTs may be best approached with allele-specific knockdown methods to try to eliminate the expression of the mutant mRNA. Piloting such approaches in CMT represents another way in which CMT translational research could have a broader impact for other rare diseases.

Induced pluripotent stem cells (iPSCs), which offer an unlimited supply of cells derived from adult patient cells, offer a unique opportunity for human disease modeling and investigation. After reprogramming, iPSCs can be differentiated into many different cell types including neurons and glia, which has led to important findings for understanding disease mechanisms and therapeutic approaches ^72– 75^. For example, the derivation of iPSCs from three GAN patients with different GAN mutations was recently reported and key pathological phenotypes observed. These cells were used to support the feasibility of gene replacement therapy ^76^. iPSC-derived motor neurons from axonal CMT patients identified common pathophysiological mechanisms in axonal CMT2A and CMT2E ^77^. Differentiation of CMT iPSCs into Schwann cells, which are most affected cell type in CMT, will be an important next step. Despite progress recapitulating Schwann cell developmental stages, improvement of differentiation protocols is still required to achieve mature, functional Schwann cells with high efficiency ^78– 80^.

## Basic research: improving collaboration and using computational approaches

We have previously described how research collaborations may enhance drug discovery and how computational approaches can be used to facilitate secure sharing of data between collaborators ^81, 82^. The NIH Blueprint Neurotherapeutics Network ^83^ is one example which provides support for small molecule drug discovery and development, access to NIH-funded contract research organizations (CROs), and access to consultants with expertise in various aspects of drug discovery and development. At the center of this is secure collaborative software so that molecules and screening data with intellectual property IP are securely shared between the groups of collaborators ^84^. With the various screening efforts undertaken for CMT1A to date ^53, 54^ perhaps it is also worth considering using computational approaches to help identify additional compounds to test using machine learning models built with the data ^85^. Also considering some of the “molecule-centric” issues in drug discovery, the potential compound libraries to be tested could be filtered before HTS for potential PAINS ^13^, false positives, aggregators, etc. As there has been considerable investment in developing chemical probes from various screens of the NIH MLSMR library, assessment by an experienced medicinal chemist has created a score which has been used to derive a computational model ^86^. This could also be used to provide some idea that the molecule may also be reasonable to a medicinal chemist’s perspective.

## Clinical research: CMT biomarkers and natural history of disease

New clinical biomarkers are needed for CMT. Recently a novel magnetization transfer ratio (MTR) MRI assay of the proximal sciatic nerve (SN) was developed as a potential biomarker of myelin content changes in patients with CMT diseases ^87^. The relationship between MTR and clinical neuropathy scores was assessed. Mean volumetric MTR values were significantly decreased in the SN of patients with CMT1A and CMT2A relative to controls. Skin derived mRNA expression also holds promise to serve as biomarkers in CMT1A patients ^61^.

The international Inherited Neuropathy Consortium (INC) recently analyzed clinical and genetic data from 1652 patients evaluated at 13 INC centers ^88^. The disease burden of all the mutations was assessed by the CMT Neuropathy Score (CMTNS) and CMT Examination Score (CMTES). Five subtypes of CMT (CMT1A/ *PMP22* duplication, CMT1X/ *GJB1* mutation, CMT2A/ *MFN2* mutation, CMT1B/ *MPZ* mutation, and hereditary neuropathy with liability to pressure palsy/ *PMP22* deletion) accounted for 89.2% of all genetically confirmed mutations. The study also confirmed that patients could be uniformly assessed between international centers and provides a baseline for future clinical studies.

## Clinical research: clinical trials and outcomes research

We have recently described some of the compounds for CMT in preclinical ^52, 89^ or clinical trials. Two recent publications from Pharnext described the novel combination of three drugs called PXT-3003 and their effect on CMT1A both in the lab ^90^ and in a phase 2 clinical trial ^91^. Initially three drugs approved for other uses (baclofen, naltrexone and sorbitol) were tested separately and were shown to work better at increasing myelination in Schwann cells when combined together in the test tube (at concentrations far lower than their approved doses). The combination of the three drugs was shown to lower Pmp22 expression. In the rat model of CMT1A different measures of effectiveness suggested that PXT-3003 was also promising and likely efficacious. The very low doses of all three components would also negate any adverse side effects. The clinical trial used three dose levels of PXT-3003 in 80 adults with mild to moderate CMT1A. This trial confirmed the safety of the combination drug and the best improvement was seen at the highest dose. No adverse events were observed. Efficacy was also assessed using the CMTNS and Overall Neuropathy Limitations Scale (ONLS). While a relatively modest but statistically significant improvement was observed, this represents the most promising potential treatment to date surpassing the various clinical trials of ascorbic acid (vitamin C), which showed less change from baseline than high dose PXT-3003. There are still many gaps in understanding the mechanism of how PXT-3003 actually exerts its effect. It is hoped that looking at patients over a longer period and possibly treating them earlier before they become clinically affected by the disease, may improve their nerve conduction. PXT-3003 represents the most promising therapeutic for the disease in years but there is still a long way to go (several years at least as PXT-3003 will enter phase 3 clinical trials in 2015) before it may be more widely available as an FDA or European Medicines Agency (EMEA) approved treatment for CMT1A.

What do we need for this and other clinical trials to be successful? The dependence on relatively crude outcome measures like the six minute walking test etc. which are still relied on need updating with technologies which track the patient continually ^92, 93^, rather than during a visit to the neurologist. New measures or biomarkers could perhaps be developed and applied in clinical trials for CMT ^94^. In addition, more subtle analysis based on surveying the patient and the caregiver may also be instructive.

For example, if we are to learn more about CMT and the effectiveness of rehabilitation it is worth involving the patient and their caregiver. A recent Italian study described a survey of CMT patients and caregivers and their perspectives and perceptions of rehabilitation efficacy and needs ^95^. This cross-sectional study used several standard questionnaires to survey 123 patients enrolled through clinical and genetic testing. Not surprisingly, the results suggested that patients believe it is important to feel better after physical therapy. There was also a discrepancy between the perception of benefit from rehabilitation for the patient, and the caregiver’s perception of benefit. Such questionnaires might be a useful addition to clinical trials to justify a treatment approval.

Patient reported outcomes are instrumental in getting drugs approved and we are seeing an increase in drugs approved just on quality of life (QoL) ^96, 97^. One hundred and eighty-nine CMT1A patients were recently reported to have a QoL that was significantly worse versus the standard population, albeit slightly better than patients with multiple sclerosis ^98^. In this study women had earlier CMT1A onset, higher deterioration of the QoL and when assessed by the ONLS, higher disability of the upper limb ^98^. A recent study of pain in 176 children with CMT used QoL outcomes and other clinical assessments ^99^. It was shown that increased pain correlated with deteriorating QoL scores but not with more severe neuropathy. What could we do to improve QoL of patients with CMT? Clearly, in children steps to alleviate pain would be an improvement.

## Conclusion

We have briefly summarized some of the developments in CMT research of the last few years. This follows on from a meeting organized by HNF to seek different perspectives which could help us understand what areas may need support and further research. We have also attempted to prioritize areas of CMT research based on the need to bring treatments to market for the waiting patients ( Table 2).

**Table 2.** Long and short term impacting priorities for CMT research.

Priority Level	Projects
Highest	Ensure clinical trials for potential treatments for CMT1A are successful and drugs are approved rapidly by regulators. (Short term impact)
	Develop sensitive, robust outcome measures for CMT. (Long term impact)
	Demonstrate the impact of therapeutics through a measurable impact on quality of life of CMT patients. (Short term and long term impact)
	Ensure early accurate diagnosis of patients with CMT. (Long term impact)
	Set up multiple HTS using FDA and proprietary compounds against various *in vitro* models for different CMT forms. (Long term impact)
Medium	Translate early mid stage preclinical discoveries for CMT. (Mid-long term impact)
	Prioritize promising preclinical candidates for other forms of CMT that can be quickly assessed for efficacy in multiple *in vivo* animal models. (Long term impact)
	Foster increased academic-industry-foundation collaborations. (Long term impact)
	Identify new mechanisms and targets for treatment of CMT. (Long term impact)
	Recruit patients through registries for future clinical trials. (Short term impact)
Lower	Provide research materials and models in central repositories. (Long term impact)
	Explore gene therapy and stem cells as longer term approaches. (Long term impact)

Currently, CMT1A is the only type of CMT for which there is a therapeutic in clinical trials ^90, 91^. We need to ensure that this compound is successful because it will benefit the patient and CMT focused scientific community. In order to do this we may want to identify, develop and validate more robust outcome measures for CMT as described earlier. In addition to show the value of treatments and impact on the QoL of patients for insurance companies who would reimburse treatment we need more studies that look into this. Based on our priorities of helping to translate treatments to the clinic and help patients, we perhaps could focus on the development of new clinical endpoints and any efforts to improve and quantify the impact of CMT on patient QoL. If we are to impact patients with CMT it is important to diagnose their disease early and to do this accurately.

High to medium level priorities include efforts to find new treatments for other forms of CMT. This may require more collaborations to identify molecules for progression. Encouraging companies to collaborate is also in the best interests of the field but not without its complexities. We can be prepared for this by using software that enables secure collaboration between different parties earlier.

Certainly we still have only a partial understanding of CMT and the search for new mechanisms and targets would be important perhaps for future breakthroughs in treatment. Another important priority is to include recruiting more patients for future clinical trials as we may be underestimating the number of patients with CMT. Registries such as GRIN ^8^ may help in this regard to both identify patients and understand their disease and treatment needs.

If we are to encourage more research on CMT, animal and cell models, chemicals and reagents need to be available readily and if possible centralized in a repository. Several animal and stem cell models have recently been developed, and it will be important to provide a repository so that other researchers can access them. Knowing which animal and cell models are valid and which are not could help to prevent costly clinical trials (as described for the ALS example earlier). While CMT is not a wide-spread disease and the funds available are limited, we have still to consider the development of such a repository for scientists because it would facilitate compound testing, and such repositories have been successfully developed for other diseases. As compounds like PXT-3003 progress through clinical trials and hopefully show an impact on the disease, other companies will likely show an interest in CMT. These companies will want to screen their libraries in cell and animal models. As we have seen in recent years, preclinical research is happening through collaboration with academic screening centers ^100^. It will be important therefore that as a community we fully characterize these systems and have them available for testing.

We should also be open to considering biotherapeutic approaches which could complement small molecule drugs. It would appear that there has been relatively little research in gene therapy for CMT. We could certainly leverage the considerable developments that have been occurring for other diseases in this regard. As a small rare disease foundation we have to consider carefully where we put our resources.

Clearly there are many other fascinating areas of research that are ongoing or may be required. The clinical trials for CMT recently undertaken are using relatively crude measures for determining efficacy. Longer-term assessments of patients using proteomic and metabolomic approaches may help to identify new biomarkers for CMT. In addition, the development of new scores for the disease ^101^ as well as standardizing them across institutions is important. Developing and accessing approaches that offer a real-time readout on the disease in patients would also be valuable for future clinical trials.

From the diagnostic side it would appear that assessing just four genes including *PMP22* duplication/deletions, *GJB1, MPZ,* and *MFN*, would capture most of the patients with a CMT phenotype. If we are to simplify diagnosis and perhaps reduce costs, a more simplistic algorithm is needed. What other factors may be important for CMT that could modify the disease or the QoL? For example a recent study of meal frequency from studies of animal and human subjects suggests that intermittent energy restriction can improve health indicators and counteract disease processes ^102^. These changes in meal frequency could have an impact on overall health. Should we be studying the effect of this intermittent energy restriction on specific diseases like CMT also?

In summary, we have drawn attention to some of the most recent advances in CMT research and made suggestions of where funding bodies such as HNF could invest to have maximum short term impact (e.g. ensuring a treatment for CMT1A is approved quickly), as well as long term impact (e.g. prioritizing compounds for other forms of CMT) ( Table 2). Our hope is that once a treatment for CMT1A is approved more drug companies will be interested in CMT, investment in research will increase and therefore we have to be prepared for that and the downstream implications on resources, research materials, researchers and ultimately patients themselves.
