# Supplementary material for: A brief review of recent Charcot-Marie-Tooth research and priorities
Source: F1000Res. 2015 Feb 26;4:53. [Version 1] doi: 10.12688/f1000research.6160.1 (PMC4392824; doi:10.12688/f1000research.6160.1)
Supplement: Peer review history

# Peer review: Referee response for version 1

**DOI:** 10.5256/f1000research.6604.r7845

**Published:** 2015-04-01

**Authors:** Gerard Said

**Affiliations:** 1 Department of Neurology, Hôpital Pitié-Salpêtrière, Paris, 75013, France

The authors discuss the many issues that can complicate the discovery of new drugs.

The authors summarize recent developments in Charcot-Marie-Tooth disease which may be used as an example of what can or should be done to improve development of drugs in rare disease.

The vast majority of CMT patients have copy number variations of *PMP22. *The authors propose a method destined to accelerate the identification of the different mutations associated with the CMT phenotype.

The authors briefly review the treatment used in CMT1A rat models and consider their application to humans.

Recently association of small doses of baclofen, naltrexone and sorbitol (PXT-3003) seemed promising and well tolerated. Phase 3 study is going to start in 2015, and it will take several years before a significant effect of the medication on CMT1A can be confirmed and the drug approved for treatment.

The different methods of evaluation of the benefit of treatment are discussed. In addition, it must be kept in mind that CMT1A is a very slowly progressive disease, with phenotypic variations especially in presentation and course. A large number of patients carrying the same mutation thus need to be included in the studies and followed over many years. It will not be an easy task in a rare disease.

This paper is useful in delineating the different steps that can lead to discovery of new drug in rare diseases, emphasizing the need for collaboration between geneticists, biochemists, drug companies  and clinicians

I have read this submission. I believe that I have an appropriate level of expertise to confirm that it is of an acceptable scientific standard.

# Peer review: Referee response for version 1

**DOI:** 10.5256/f1000research.6604.r7842

**Published:** 2015-03-17

**Authors:** Rhona Mirsky

**Affiliations:** 1 Department of Cell and Developmental Biology, University College London, London, UK

This report reviews recent research on Charcot-Marie-Tooth neuropathies. The report is the result of a recent meeting of the scientific advisory board of the Hereditary Neuropathy Foundation and summarises suggestions made there. The article includes suggestions for improvements to diagnosis, including stratified testing for the most common mutations before screening for the rarer ones, and discussion of new and old animal models that have been developed to study and understand disease mechanisms and for the exploration of possible therapeutic strategies. It reviews other avenues of active research, including selective use of adenoviral vectors and stem cells that are being investigated for possible use in gene therapy for some forms of the disease, for understanding disease mechanisms, and for screening programmes for therapeutic drugs. It highlights the possibility that chemotherapy-induced toxicity may exacerbate some forms of CMT. It suggests that there is room for improved collaboration between different research groups and clinicians and more use of computational approaches. To my knowledge there are already several consortia in addition to the Hereditary Neuropathy Foundation that are already very active in trying to advance therapeutic strategies so I am not sure how much of a deficit there is in this area.  On the clinical side a need for reliable biomarkers of the various disease forms is highlighted and the review ends with a discussion of clinical trials and the importance of involving patients and caregivers in the trial outcome measures is emphasised.

Overall the article provides an up to date review of the research and possible treatments for this group of relatively rare diseases which will be of use to both researchers and clinicians in the field.

I have read this submission. I believe that I have an appropriate level of expertise to confirm that it is of an acceptable scientific standard.
